# Influence of Removing Leaf Node Neighbors on Network Controllability

**DOI:** 10.3390/e25060945

**Published:** 2023-06-15

**Authors:** Chengpei Wu, Siyi Xu, Zhuoran Yu, Junli Li

**Affiliations:** 1 School of Computer Science, Sichuan Normal University, Chengdu 610068, China; chengpei.wu@hotmail.com (C.W.); xusiyi1999@163.com (S.X.); yuzhuor@foxmail.com (Z.Y.); 2 Visual Computing and Virtual Reality Key Laboratory of Sichuan, Sichuan Normal University, Chengdu 610068, China

**Keywords:** controllability, controllability robustness, undirected networks, attack strategy

## Abstract

From the perspective of network attackers, finding attack sequences that can cause significant damage to network controllability is an important task, which also helps defenders improve robustness during network constructions. Therefore, developing effective attack strategies is a key aspect of research on network controllability and its robustness. In this paper, we propose a Leaf Node Neighbor-based Attack (LNNA) strategy that can effectively disrupt the controllability of undirected networks. The LNNA strategy targets the neighbors of leaf nodes, and when there are no leaf nodes in the network, the strategy attacks the neighbors of nodes with a higher degree to produce the leaf nodes. Results from simulations on synthetic and real-world networks demonstrate the effectiveness of the proposed method. In particular, our findings suggest that removing neighbors of low-degree nodes (i.e., nodes with degree 1 or 2) can significantly reduce the controllability robustness of networks. Thus, protecting such low-degree nodes and their neighbors during network construction can lead to networks with improved controllability robustness.

## 1. Introduction

Complex networks have developed rapidly over the past two decades [1]. There are many real-world systems that can be modeled as complex networks, with a large number of nodes and edges. The study of networks can help us understand corresponding complex systems. For example, social networks can help us understand the ways in which humans interact and how information spreads in society; transportation networks can help us study the flow and congestion of traffic in cities. The controllability of a network is a crucial factor, as control networks are designed to serve people [2,3]. In this context, controllability refers to the ability of a dynamical network to be guided by external inputs from any initial state to any desired target state within a finite duration of time under an admissible control input.

Recently, failures and attacks on complex networks have become more frequent and severe [4,5,6,7,8]. When failures and attacks occur, they are removed in the form of node or edge removals. On node removals, the target node and the edges previously connected to the node will be removed, while on edge removals, only the target edges will be disconnected. Network attacks are typically categorized into two types: random and malicious. Random attacks refer to the uniform random selection of attack targets, while malicious attacks choose the most effective targets to attack. Malicious attacks typically have a better effect than random attacks, but they also consume more computation time. During malicious attacks, the most destructive target is selected for attacking; such target choices are usually based on the centrality of nodes. For example, the node with the highest degree is attacked firstly or the edge with the highest betweenness is preferentially removed. Besides degree and betweenness, commonly used measures of node importance include closeness [9], Katz centrality [10], neighborhood similarity [11], branch weighting [12], structural holes [13], and so on. Many malicious attack models have been proposed; the hierarchical structure of a directed network enables a random upstream (or downstream) attack on the network controllability, which results in a more destructive attack strategy than random attacks [14]. In addition, the module-based attack strategy [15,16] aims at attacking the nodes with inter-community edges that are crucial to maintain connectivity among communities.

In addition, nodes can be categorized as critical or non-critical based on their impact on network controllability when removed. Removing critical nodes can significantly reduce network controllability [6], so they should be protected during network construction to enhance controllability robustness. Bridges, a type of edge in networks, can also be targeted for removal to decrease network controllability [17]. Many deep learning and optimization methods can be used to find such hidden patterns and key roles of networks; the proposed Finder framework, which employs reinforcement learning, offers a unified approach for identifying a group of nodes that can destroy network function to the greatest extent after removals [18]. Deep learning models are used to predict network robustness [19,20,21,22]; evolutionary algorithms are utilized for network attacks [23,24].

The research on network attacks aims to enhance the robustness of networks from the perspective of attackers, enabling them to better withstand attacks when constructing networks. One effective approach to improving the controllability robustness of networks is to protect bridges, as they play a critical role in network connectivity. Protecting critical nodes and edges during network construction is also a viable strategy for enhancing controllability robustness [6]. Studies have shown that three-ring and four-ring structures in networks are beneficial for controllability robustness [25], which suggests that networks with random triangle and random quadrilateral structures tend to exhibit good controllability robustnesss.

The structural controllability of the network can be evaluated by identifying the maximum matching of the network to determine the minimum number of external control inputs required as driver nodes [4]. However, this approach is only suitable for directed networks and is challenging to apply to large-scale networks. To address this issue, the exact controllability framework was proposed, which can be utilized for all large-scale sparse networks [26].

Controllability robustness can be measured using two approaches: *A priori* measure and *A posteriori* measure. The *A priori* measure calculates network features in a single calculation, while the *A posteriori* measure simulates the change in network controllability curve under attack. Although the *A posteriori* measure is more accurate, it can be computationally expensive, especially for large-scale networks. Recently, deep learning has emerged as a promising approach to measure network robustness accurately and efficiently, providing a less time-consuming alternative to simulation-based methods [19,21,22,27,28].

The selection of literature reviewed in this paper was based on the following criteria: (1) Relevance to the research objective: we screened literature that directly related to our research topic. (2) Credibility and authority: we evaluated the credibility and authority of the literature. We specifically focused on articles published in reputable journals, conference proceedings, and reports from authoritative research organizations. (3) Time range: we limited the articles to recent publications as much as possible to ensure they represented the latest advancements in the field. By employing these criteria, we aimed to ensure the selection of the most relevant, credible, and recent literature to support the research objectives.

Overall, this paper presents an attack strategy for undirected network controllability. The main contributions are:

(1) A novel attack strategy is proposed, which can effectively disrupt the controllability of undirected networks.
(2) The impact of removing nodes with degree 1 and 2 on network controllability is analyzed, revealing that nodes with low degree are not beneficial to the robustness of network controllability.
(3) The findings provide valuable insights for identifying key nodes and designing networks with improved controllability robustness in future research.

The rest of this paper is organized as follows: Section 2 introduces the preliminary concepts of controllability and controllability robustness; Section 3 illustrates in detail the proposed attack model LNNA; Section 4 demonstrates the experimental results on synthetic and real-world networks; and Section 5 concludes this paper.

## 2. Network Controllability and Controllability Robustness

The robustness of network controllability is mainly concerned with the change in controllability when the network is attacked [29]. Controllability robustness reflects the ability of the network to resist attacks, from the perspective of attackers, which can also be the evaluation index of attack performance, namely, the worse the robustness, the better the attack model. Network controllability is measured by the density of driver nodes $n_{D}$,

(1) $$n_{D}=\frac{N_{D}}{N}$$

where $N_{D}$ represents the number of driver nodes required to maintain network controllability and *N* represents the total number of network nodes. The minimum value of $n_{D}$ is $\frac{1}{N}$, and the maximum value is 1. A smaller value of $n_{D}$ indicates better network controllability, while a larger value of $n_{D}$ indicates worse network controllability. According to the minimum-inputs theorem [4], for directed networks, $N_{D}$ can be obtained by the number of unmatched nodes for a directed network:

(2) $$N_{D}=\max\left\{1,N-\left|E^{*}\right|\right\}$$

where $|E^{*}|$ is the size of maximum matching. As for exact controllability [26], $N_{D}$ is calculated by:

(3) $$N_{D}=\max\left\{1,N-rank\left(A\right)\right\}$$

where rank(A) is the rank of the adjacency matrix *A*. For node-removal attacks, the controllability robustness of the network can be measured by the controllability curve, which is calculated as follows:

(4) $$n_{D}\left(i\right)=\frac{N_{D}\left(i\right)}{N-i},i=0,1,\ldots ,N-1,$$

where $N_{D}\left(i\right)$ the number of driver nodes needed to maintain network controllability after removing a total of *i* nodes, *N* is the size of the original network, $N_{D}$ is calculated by using the exact controllability framework as Equation (3), which applies to large and sparse undirected networks, $n_{D}$ records the changes in network controllability after each node is removed. The overall measure of controllability robustness can be obtained by averaging controllability curves as follows:

(5) $$R_{N}=\frac{1}{N-1}\sum_{i=1}^{N-1}n_{D}\left(i\right),$$

where $n_{D}\left(i\right)$ is the structural controllability of the remaining network after *i* nodes are removed. The network controllability robustness can be evaluated by $R_{N}$, a smaller $R_{N}$ indicates better controllability robustness, while a larger $R_{N}$ indicates worse controllability robustness. For an attack model, a better controllability robustness indicates a worse attack performance.

## 3. Leaf Node Neighbor-Based Attack Strategy

The removal of different nodes in networks has different impacts on the controllability of networks. Leaf nodes, which are the nodes with a degree of 1, are most likely driver nodes in the network. If the only neighbor of a leaf node is removed, the leaf node will become an isolated node and the number of connected components of the network will increase. Furthermore, after the neighbor of a leaf node is attacked, which is connected to many leaf nodes, all the leaf nodes connected to it will become drive nodes. Therefore, the neighbors of a leaf node are important for controllability attacks, and the number of leaf nodes connected by that neighbor node is also an important reference. The above ideas are applied to the following attack strategy design; the following subsections analyze in detail the impacts of removing leaf node neighbors on network controllability.

### 3.1. Leaf Node Neighbor-Based Attack Strategy

As neighbors of leaf nodes can be removed to reduce the controllability of a network, a leaf node neighbor-based attack (LNNA) strategy is proposed. Before proposing the algorithm, there are two necessary concepts, *k*-neighbor node and *k*-neighbor degree, which are pre-defined, where *k*-neighbor node is a neighbor of a node with degree *k*, and *k*-neighbor degree for a node is the number of neighbors with degree *k*. The proposed LNNA is based on the above concepts and is described in detail in Algorithm 1.
**Algorithm 1** Leaf Node Neighbor-based Attack Strategy.**Input:** a network *G* with *N* nodes
**Output:** index *t* of target node to be attacked
  1: degrees←{}
  2: neighbors←{}
  3: // get node degrees and neighbors
  4: **for** i=1 to *N* **do**
  5:   $d\leftarrow get\_degree(node_{i})$
  6:   **if** d!=0 **then**
  7:     degrees[i]←d
  8:      $neighbors\left[i\right]\leftarrow get\_neighbor\_indexes\left(node_{i}\right)$
  9:   **end if**
10: **end for**
11: **if** degrees.size()!=0 **then**
12:   // get the nodes and their neighbors with smallest degree *k*, generally, *k* equals to 1
13:   k←degrees.minimum()
14:   k_nodes←{}
15:   k_neighbors←{}
16:   **for** j=degrees.keys() **do**
17:     **if** degrees[j]==k **then**
18:        k_nodes.insert(j)
19:        k_neighbors.insert(neighbors[j])
20:     **end if**
21:   **end for**
22:   k_neighbor_degrees←Array(k_neighbors.size())
23:   **for** j=1 to k_neighbors.size() **do**
24:     **for** neighbor_id=get_neighbor_indexes(k_neighbors[j]) **do**
25:        **if** neighbor_id in k_nodes **then**
26:           k_neighbor_degrees[j]←k_neighbor_degrees[j]+1
27:        **end if**
28:     **end for**
29:   **end for**
30:   t←k_neighbors[argmax(k_neighbor_degrees)]
31: **else**
32:   t←random_integer(1,N)
33: **end if**
34: **return** *t*


As shown in Algorithm 1, LNNA usually starts from the neighboring node of leaf nodes, i.e., 1-neighbors. When attacking the 1-neighbors, the attack target is determined based on the number of leaf nodes connected to these nodes, i.e., the 1-neighbor degree. The node with the highest 1-neighbor degree among the 1-neighbors will be attacked. When there are no leaf nodes in the network, LNNN will choose nodes to attack from the 2-neighbors. Similarly, the node with the highest 2-neighbor degree among the 2-neighbors will be selected as the target. This process continues recursively.

In terms of time complexity, for each attack, finding neighbors of each node is $O(N^{2})$, *N* is the number of nodes; finding and getting the smallest *k*-neighbors is O(N); getting *k*-neighbor degrees is O(KN), *K* is the number of *k*-neighbors, K<N; thus, the total time complexity is $\max\left\{O\left(N^{2}\right),O\left(N\right),O\left(KN\right)\right\}=O\left(N^{2}\right)$. For typical malicious attack methods, namely, degree- (DEG), betweenness- (BET), closeness- (CLO) based attacks, the time complexity comparison is listed in Table 1. For degree-based attacks, getting the degree of each node is $O(N^{2})$, similar to finding neighbors of each node; for a betweenness-based attack, getting the betweenness of each node is $O(N^{3})$ when using the Floyd–Warshall algorithm; for a closeness-based attack, computing time of closeness is O(N(M+N)), and *M* is the number of edges.

### 3.2. Influence of Leaf Node Neighbor Failures

An algorithm that targets the neighbors of low-degree nodes (i.e., leaf nodes) with a node attack has been developed in Section 3.1. In this subsection, we also provide a mathematical proof detailing why attacking the neighbors of leaf nodes is effective and leads to an increase in the number of driver nodes.

**Theorem** **1.** *Let $A_{N}$ be the adjacency matrix of a network, and $A_{N-1}$ be the adjacency matrix after removing a node $V_{r}$. $rank\left(A_{N}\right)=rank\left(A_{N-1}\right)+2$, if the following condition holds: $V_{r}$ is the only neighbor of a leaf node $V_{l}$.*

**Proof.**  The adjacency matrix $A_{N}$ and $A_{N-1}$ can be represented as follows:

$$A_{N}=\left[\begin{array}{ccc} 0 & \vec{0^{\prime }} & 1\\ \vec{0} & D & \vec{a^{\prime }}\\ 1 & \vec{a^{\prime }} & 0 \end{array}\right],A_{N-1}=\left[\begin{array}{cc} 0 & \vec{0^{\prime }}\\ \vec{0} & D \end{array}\right],$$

As the permutation invariance of adjacency matrix, the first row (column) of $A_{N}$ represents $V_{l}$, the last row (column) of $A_{N}$ represents $V_{r}$; it is clear that

$$rank\left(D\right)=rank\left(A_{N-1}\right)=rank\left(A_{N}\right)-2$$

□

**Theorem** **2.** *Let $N_{D}$ be the number of driver nodes in a network, and $N_{D}^{{}^{\prime }}$ be the number of driver nodes on the network after removing a leaf node neighbor. $N_{D}^{{}^{\prime }}=N_{D}+1$, if the following condition holds: $rank(A_{N})<N$.*

**Proof.**  If $rank(A_{N})<N$, then, $rank\left(A_{N-1}\right)=rank\left(A_{N}\right)-2<N-2$. Therefore, the following equation holds:

$$\begin{array}{cc} N_{D} & =\max\{1,N-rank\left(A_{N}\right)\}\\ \; & =N-rank(A_{N})\\ \; & =N-rank(A_{N-1})-2\\ \; & =[\left(N-1\right)-rank\left(A_{N-1}\right)]-1\\ \; & =\max\{1,\left(N-1\right)-rank\left(A_{N-1}\right)\}-1\\ \; & =N_{D}^{{}^{\prime }}-1 \end{array}$$

□

**Theorem** **3.** *Let $N_{D}$ be the number of driver nodes in a network, and $N_{D}^{{}^{\prime }}$ be the number of driver nodes on the network after removing a leaf node neighbor. $N_{D}^{{}^{\prime }}=N_{D}=1$, if the following condition holds: $rank(A_{N})=N$.*

**Proof.**  If $rank(A_{N})=N$, then $N_{D}=max\left\{1,N-rank\left(A_{N}\right)\right\}=1$, the following equation holds:

$$rank\left(A_{N-1}\right)=rank\left(A_{N}\right)-2=N-2,N_{D}^{{}^{\prime }}=max\{1,\left(N-1\right)-rank\left(A_{N-1}\right)=1$$

□

According to Theorem 1, 2, and 3, removing the neighbor of a leaf node from the network increases or maintains the total number of driver nodes. From Equations (1) and (4), we can find that the more driver nodes there are, the worse controllability the network has.

## 4. Experimental Studies

LNNA is applied to three kinds of synthetic networks and real networks. In order to verify its effectiveness, the performance of LNNA was compared with feature-based attacks such as degree-, betweenness-, and closeness-based attacks. The main focus of this paper is to investigate the impact of attacking nodes with the lowest degree, excluding isolated nodes, on network controllability. Specifically, the study explores the effects of removing neighbors of leaf nodes on network controllability.

The three synthetic networks are Erdös-Rényi (ER) random-graph [30], Generic scale-free (SF) network [31], and Newman–Watts small-world (SW) network [32]. The real-world networks are *econ-mahindas* and *soc-wiki-Vote*; all networks are undirected. The number of nodes are N=500 and N=1000; the average degree 〈k〉=3,5, and 10, respectively. In order to reduce the influence of randomness, 30 random instances are generated for each network.

### 4.1. Results on Synthetic Networks for Different Average Degrees

Liu et al. [4] suggested that controlling sparse heterogeneous networks can be challenging and that such networks tend to have poor network controllability and robustness. Conversely, dense homogeneous networks are easier to control and have better network controllability and robustness. Moreover, networks with higher average degrees have more redundant edges, which help maintain the core structure of the network and make it less susceptible to destruction, resulting in better network controllability and robustness.

**Figure 1:**
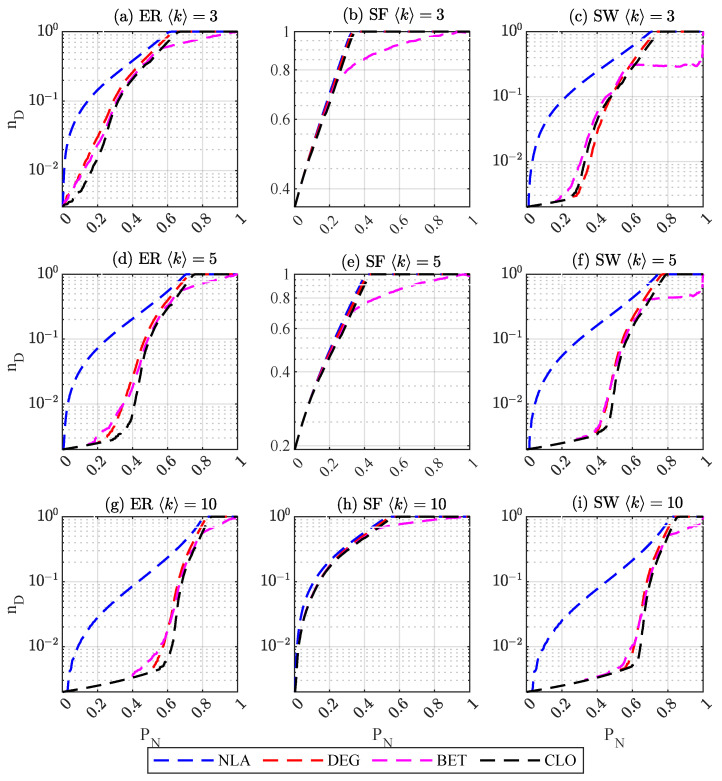
Controllability robustness of networks with N=1000.

As shown in Figure 1, for ER and SW networks with an average degree of 3, LNNA can effectively disrupt network controllability from the beginning of attacks. When the average degree is 5, for the degree- (DEG), betweenness- (BET), closeness- (CLO) based attacks on ER networks, the controllability of ER networks begins to decrease significantly after about 20% of nodes are removed, and for the SW networks, the threshold proportion is 40%. However, LNNA can significantly reduce the controllability of networks from the beginning of the attacks. For SF networks, there is no significant difference between different attacks, since the topology of SF networks makes the network robust on controllability, and nodes that can disrupt the network controllability are easy to find. When 〈k〉=10, for ER networks, the three feature-based attacks rapidly reduce the controllability of the network when more than 40% of the nodes are removed. For SW networks, the ratio is around 50%. For SF networks, LNNA is slightly better than other attacks, because with the increase in average degree, the network controllability robustness is improved. Overall, as shown in Table 2, LNNA has the best attack effect on the three synthetic networks with different average degrees.

### 4.2. Results on Synthetic Networks for Different Network Sizes

**Figure 2:**
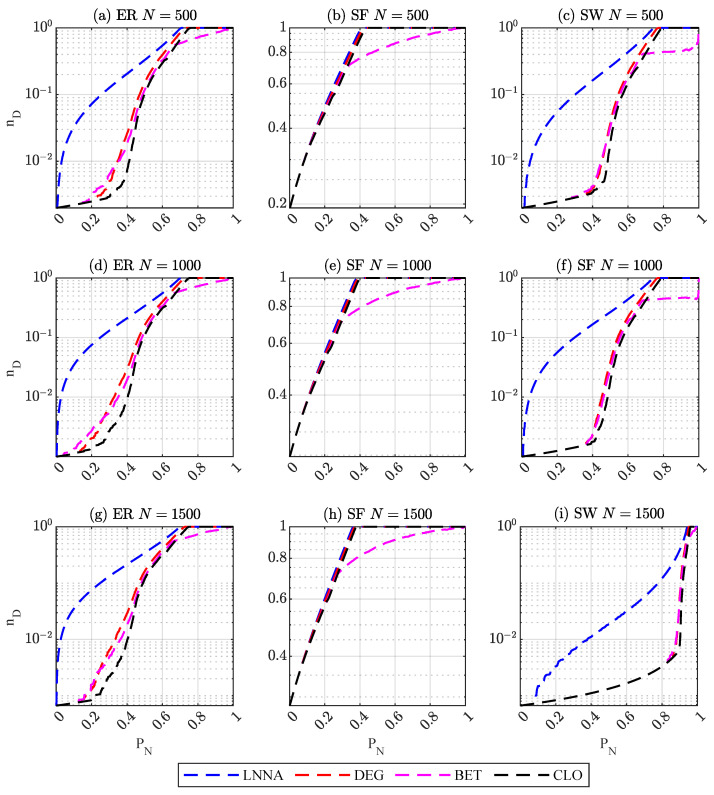
Controllability robustness of networks with 〈k〉=5.

The size of a network is typically defined as the number of nodes in the network. Large-scale networks tend to be more complex and may exhibit a variety of structures, and the controllability robustness of networks can vary across different scales. Moreover, in terms of attack strategies, networks with more nodes require higher computational costs relative to smaller networks. As shown in Figure 2, the effect of different attacks on the three synthetic networks is similar when the average degree is 5, regardless of network size (i.e., 500, 1000, or 1500 nodes). However, LNNA shows better attack performance on ER and SW networks compared to other attack methods, while no significant difference is observed in SF networks. Table 3 also shows that when the average degree is 5, LNNA performs the best on the three networks with 500, 1000, and 1500 nodes.

### 4.3. Results on Real-World Networks

LNNA is applied to two real-world networks, called *econ-mahindas* and *soc-wiki-Vote* [33]. The information of networks is shown in Table 4.

**Figure 3:**
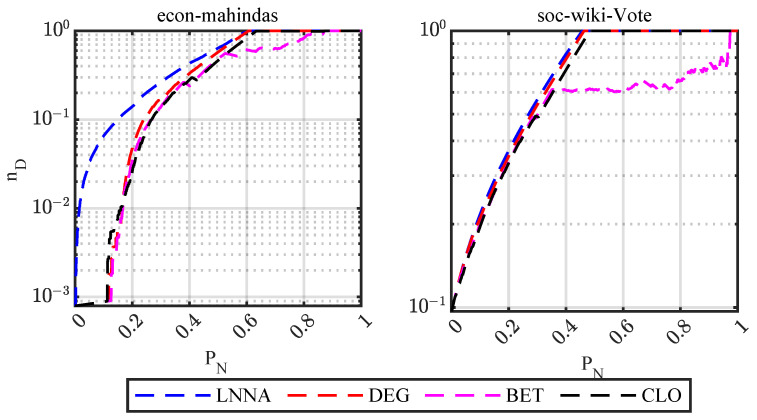
Controllability robustness of real-world networks.

As shown in Figure 3, for the *econ-mahindas* network, the attack effect of LNNA is obviously better than that of other attack strategies. For the *soc-wiki-Vote* network, the attack effect of LNNA is slightly better than other strategies, but there is no significant difference. These results are consistent with those obtained on synthetic networks. Furthermore, for networks with good controllability robustness, LNNA is more destructive to the network controllability.

### 4.4. Attack Process Discussion

**Figure 4:**
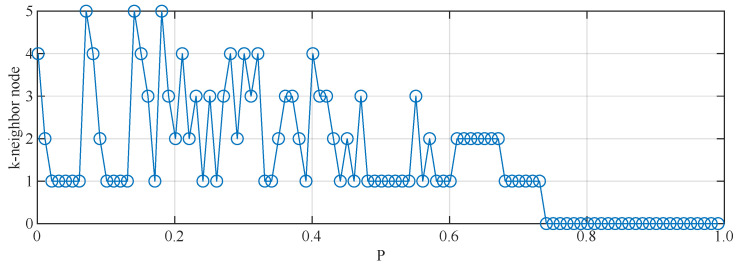
Visualization of targeted nodes in the attack process of LNNA.

Figure 4 illustrates types of the selected nodes during the LNNA. When approximately 70% of the nodes are removed, only isolated nodes remain in the network.

**Figure 5:**
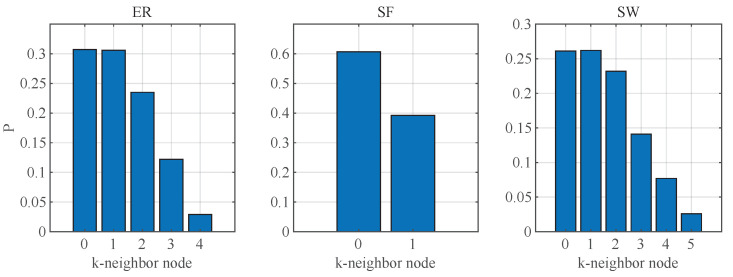
The proportion of attacked node types in the three networks under LNNA, N=1000 and 〈k〉=5.

Figure 5 illustrates the proportion of different nodes removed by different networks under LNNA. It can be observed that the proportion of nodes decreases as the *k* of *k*-neighbor increases. Apart from isolated nodes, the largest proportion represents 1-neighbor nodes, which refers to the neighbor of a leaf node. When a *k*-neighbor node is attacked, a (k−1)-neighbor node is generated. In the case of ER and SW networks, 1-neighbor nodes account for approximately 30% and 25%, respectively, and the damage to network controllability is significant. For the SF network, it can be seen that the attacked nodes are 1-neighbor nodes, accounting for about 40%. Subsequently, only isolated nodes remain in the network, indicating that the SF network is vulnerable to attack.

## 5. Conclusions

In order to investigate the controllability robustness of networks from the perspective of attacks, this paper mainly examines the destructive impact of the neighbor nodes of a node with low degree on network controllability. We proposed an attack strategy targeted on leaf node neighbors, named LNNA, and introduced the defined concepts of *k*-neighbor nodes and *k*-neighbor degrees. First, we investigated the role of neighboring nodes of leaf nodes in network controllability and the changes that occur in network controllability when these nodes are removed. Then, we defined the concepts of *k*-neighbor nodes and *k*-neighbor degrees, which denote the neighboring nodes of a node with degree *k*, and the degree of *k*-neighbor node itself. Based on the exploration, we proposed an attack strategy targeting these nodes to achieve maximum disruption of network controllability; the attack strategy prioritizes attacking the neighbor node of a node with the lowest degree, except for isolated nodes. In a network, if the *k*-neighbor nodes are attacked, the (k−1)-neighbor nodes will be generated, and this process continues until 1-neighbor nodes appear, which will then be attacked. Simulated experiments on synthetic and real-world networks demonstrate that the proposed LNNA performs better than degree-, betweenness-, and closeness-based attacks. This suggests that the presence of low-degree nodes in networks is not conducive to network controllability robustness. In the future, when designing networks with good controllability robustness, it is advisable to make the network more homogeneous to avoid the presence of a large number of low-degree neighbor nodes, such as 1-neighbor nodes and 2-neighbor nodes.

## Figures and Tables

**Table 1:** Time complexity of attack methods.

Method	LNNA	DEG	BET	CLO
Time complexity	$O(N^{2})$	$O(N^{2})$	$O(N^{3})$	O(N(M+N))

**Table 2:** Robustness of network controllability with different average degrees; the values are calculated by Equation (5).

Networks	〈k〉	LNNA	DEG	BET	CLO
ER	3	0.5893	0.5258	0.4274	0.4921
	5	0.4759	0.3942	0.3139	0.3619
	7	0.3382	0.2537	0.2012	0.2299
SF	3	0.8836	0.8808	0.8259	0.8759
	5	0.8085	0.8027	0.7322	0.7938
	7	0.6636	0.6421	0.5683	0.6273
SW	3	0.4961	0.382	0.1612	0.3656
	5	0.4324	0.3354	0.1802	0.3073
	7	0.3236	0.2375	0.166	0.2173

**Table 3:** Robustness of network controllability with different network sizes; the values are calculated by Equation (5).

Network	*N*	LNNA	DEG	BET	CLO
ER	500	0.4759	0.3942	0.3139	0.3619
	1000	0.4803	0.3963	0.318	0.3654
	1500	0.4815	0.3966	0.3211	0.3676
SF	500	0.8085	0.8027	0.7322	0.7938
	1000	0.8357	0.8312	0.765	0.8242
	1500	0.8519	0.848	0.787	0.8422
SW	500	0.4324	0.3354	0.1802	0.3073
	1000	0.4347	0.3368	0.1788	0.3066
	1500	0.1238	0.072	0.0582	0.061

**Table 4:** Information of real-world networks.

Network	*N*	*M*
econ-mahindas	1258	7682
soc-wiki-Vote	889	2914

## Data Availability

Not applicable.
